# Ago-RIP-Seq identifies Polycomb repressive complex I member CBX7 as a major target of *miR-375* in prostate cancer progression

**DOI:** 10.18632/oncotarget.10729

**Published:** 2016-07-20

**Authors:** Julia M.A. Pickl, Diana Tichy, Vladimir Y. Kuryshev, Yanis Tolstov, Michael Falkenstein, Julia Schüler, Daniel Reidenbach, Agnes Hotz-Wagenblatt, Glen Kristiansen, Wilfried Roth, Boris Hadaschik, Markus Hohenfellner, Stefan Duensing, Doreen Heckmann, Holger Sültmann

**Affiliations:** ^1^Cancer Genome Research Group, German Cancer Research Center (DKFZ) and German Cancer Consortium (DKTK), Heidelberg, Germany; ^2^Department of Biostatistics, German Cancer Research Center (DKFZ), Heidelberg, Germany; ^3^Section of Molecular Urooncology, Department of Urology, University of Heidelberg School of Medicine, Heidelberg, Germany; ^4^Oncotest GmbH, Institute for Experimental Oncology, Freiburg, Germany; ^5^Bioinformatics Group, Core Facility Genomics & Proteomics, German Cancer Research Center (DKFZ), Heidelberg, Germany; ^6^Institute of Pathology, Center for Integrated Oncology, University of Bonn, Bonn, Germany; ^7^NCT Tissue Bank of The National Center of Tumor Diseases (NCT) and Institute of Pathology, University Hospital Heidelberg, Heidelberg, Germany; ^8^Department of Urology, University Hospital Heidelberg, Heidelberg, Germany

**Keywords:** *miR-375*, *CBX7*, biomarker, prostate cancer, Ago-RIP-Seq

## Abstract

Prostate cancer is a heterogeneous disease. *MiR-375* is a marker for prostate cancer progression, but its cellular function is not characterized. Here, we provide the first comprehensive investigation of *miR-375* in prostate cancer. We show that *miR-375* is enriched in prostate cancer compared to normal cells. Furthermore, *miR-375* enhanced proliferation, migration and invasion *in vitro* and induced tumor growth and reduced survival *in vivo* showing that *miR-375* has oncogenic properties in prostate cancer. On the molecular level, we provide the targetome and genome-wide transcriptional changes of *miR-375* expression by applying a generalized linear model for Ago-RIP-Seq and RNA-Seq, and show that *miR-375* is involved in tumorigenic networks and Polycomb regulation. Integration of tissue and gene ontology data prioritized *miR-375* targets and identified the tumor suppressor gene *CBX7*, a member of Polycomb repressive complex 1, as a major *miR-375* target. *MiR-375*-mediated repression of *CBX7* was accompanied by increased expression of its homolog *CBX8* and activated transcriptional programs linked to malignant progression in prostate cancer cells. Tissue analysis showed association of CBX7 loss with advanced prostate cancer. Our study indicates that *miR-375* exerts its tumor-promoting role in prostate cancer by influencing the epigenetic regulation of transcriptional programs through its ability to directly target the Polycomb complex member *CBX7*.

## INTRODUCTION

Prostate cancer is the most frequent type of cancer among males in developed countries and one of the leading causes of cancer mortality. The clinical course of prostate cancer is very heterogeneous and ranges from indolent to aggressive tumors. However, there is currently no way to safely distinguish patients who need treatment from those who do not. Therefore, it is crucial to find and characterize novel diagnostic and prognostic prostate cancer markers.

MicroRNAs (miRs) are ~19-22 nt short non coding RNAs, which are known to be regulators of gene expression. They are transcribed as primary miRs (pri-miRs) by RNA Polymerase II and are further processed to precursor miRs (pre-miRs) by DROSHA [1]. DICER1 cleaves them to mature miRs, which exert their functions by guiding the RNA induced silencing complex (RISC) with its catalytic component Argonaute (Ago) to RNAs in the cytoplasm, thereby inhibiting translation and degrading target RNA [1]. Deregulation of miRs is associated with various diseases including cancer, and it has been shown that miR abundances in tissue or serum of prostate cancer patients correlate with tumor aggressiveness [2], suggesting miRs as biomarkers for diagnosis, prognosis and response to therapy in prostate cancer [3–5].

Previously, we identified circulating *miR-375* as a progression marker of prostate cancer [2]. *MiR-375* abundance in serum of prostate cancer patients correlates with the Gleason Score and lymph node metastasis [6, 7], castration resistant metastatic prostate cancer [8], and poor prognosis [9]. In high-risk tumors, *miR-375* levels along with prostate specific antigen (PSA) improved prediction accuracy compared to PSA alone [2]. *MiR-375* has been described both as a tumor suppressor and an oncogene in various cancer entities (reviewed in [10]). However, despite the high consistency among several studies describing *miR-375* as a suitable prostate cancer biomarker, its function in this tumor remains poorly understood [6, 11–13]. Thus, further efforts are needed to appropriately characterize the role of *miR-375*.

To fully understand the role of *miR-375* in prostate cancer, it is necessary to identify its target genes. Because the accuracy of miR target prediction algorithms is low [14], and previous biochemical methods for identifying the targets of specific miRs are technically and analytically difficult [15–17], we used a combination of Ago-RIP-Seq and RNA-Seq following overexpression of *miR-375* in PC-3 prostate cancer cells. We applied a novel analysis strategy based on generalized linear models for this experimental setup and integrated tissue data and gene ontology analysis (GO), to prioritize *miR-375* target genes. We identified Polycomb complex member and tumor suppressor gene CBX7 as major *miR-375* target. CBX7 repression induced malignant progression and provides a rationale for the tumor promoting role of *miR-375 in vitro* and *in vivo*.

## RESULTS

### Ago-RIP-Seq identifies *miR-375* target genes

**Figure 1:**
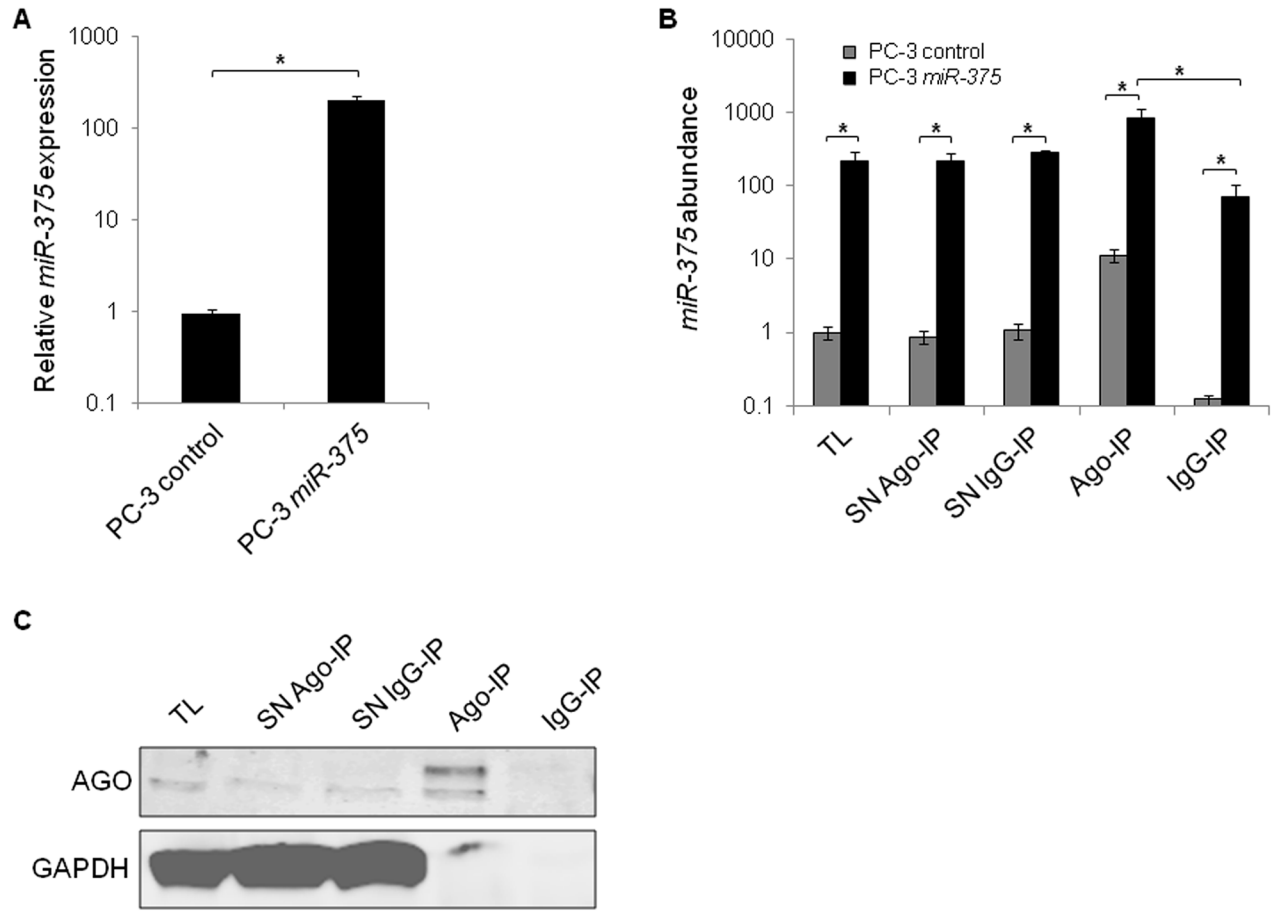
Enrichment of *miR-375* and Ago protein in Ago-RIP-Seq **A.** qRT-PCR analysis of *miR-375* expression in PC-3 *miR-375* and PC-3 control (empty vector transfection) cells. Relative *miR-375* expression was adjusted to RNU6B. **B.** *MiR-375* abundance in Ago-RIP fractions as measured by qRT-PCR. TL = total lysate, IP = immunoprecipitation, SN = IP supernatant. **C.** Specificity of Ago-IP demonstrated on western blot stained for Ago and GAPDH. Ago is pulled down, whereas GAPDH as negative control is not. All experiments were performed in three replicates. *P ≤ 0.05. All error bars show s.d.. Experiments were performed 48 h after transfection.

Functional miRs are incorporated into the Ago complex and bind to their target RNAs. To identify targets of *miR-375*, we overexpressed *miR-375* in PC-3 cells (200-fold, Figure 1A), lysed them (total lysate (TL) *miR-375* and TL control) after 48 h, and immunoprecipitated Ago complexes from total lysates with pan-Ago antibodies (Ago-IP) and from controls with isotype IgG antibodies (IgG-IP). As expected, *miR-375* was enriched in the Ago-IP fractions of PC-3 *miR-375* cells (12-fold compared to IgG-IP of PC-3 *miR-375* cells and 74-fold compared to Ago-IP of PC-3 control cells, Figure 1B). The specificity of the Ago protein pulldown was confirmed by western blot (Figure 1C). The RNA extracts of the TL, Ago- and IgG-IP fractions of three independent Ago-IP experiments were analyzed by high-throughput sequencing (Ago-RIP-Seq), that yielded between 18.9 and 28.5 (median 23.7) million reads (Supplementary Table S1, E-MTAB-3691).

**Figure 2:**
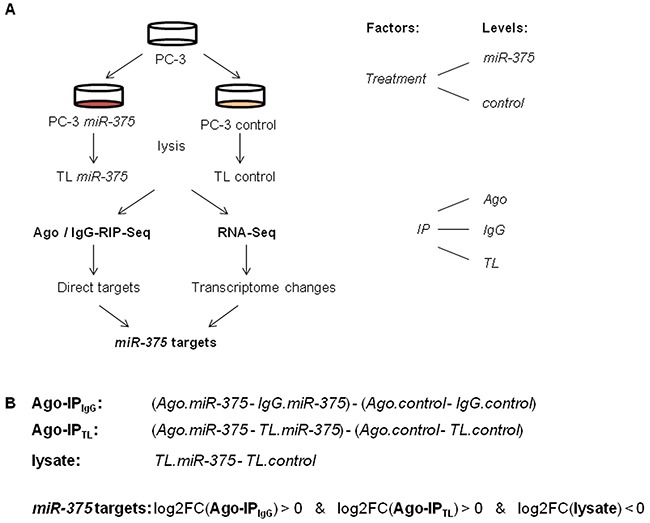
**A.** Experimental design of Ago-RIP-Seq and RNA-Seq to detect *miR-375* targets. Lysis was performed 48 h after transfection. **B.** Comparisons calculated to detect potential direct *miR-375* targets and transcriptomal changes, and definition of potential direct *miR-375* targets.

To statistically model the Ago-RIP-Seq experiment, we defined transfection of PC-3 cells with either *miR-375* or control as the factor *treatment* with the levels *miR-375* and *control*, respectively (Figure 2A). *Immunoprecipitation* (*IP*) was the second factor with the levels *Ago, IgG*, and *TL*. *IgG* and *TL* were used to adjust for background signals (Figure 1B). We then analyzed this two-factorial setup by fitting a generalized linear model (GLM) to the experimental design. We defined the comparisons Ago-IP_IgG_ and Ago-IP_TL_ to identify potential direct *miR-375* targets (Figure 2B). As target expression is known to be reduced by miRs, in parallel, we also compared these RNA profiles with transcriptome changes after *miR-375* overexpression using RNA-Seq (comparison lysate, Figure 2B, Supplementary Table S2). Potential *miR-375* targets were defined by positive log2FC values in Ago-IP_IgG_ and Ago-IP_TL_ and negative log2FC in lysate (Figure 2B). The integration of Ago-RIP-Seq and RNA-Seq data yielded 3071 potential direct *miR-375* targets (Supplementary Table S3), which is in accordance with other genome-wide miR target studies [18].

### Global analysis identifies genes regulated by the Polycomb repressive complex and tumor-promoting processes as major *miR-375* targets

To globally investigate gene regulation by *miR-375*, we conducted gene set analysis (GSA) using the PIANO algorithm [19]. The gene sets BENPORATH_ES_WITH_H3K27ME3 (“H3K27me3 marked”) [20], BENPORATH_PRC2_TARGETS (“PRC2 targets”) [20], and KRAS.PROSTATE_UP.V1_DN (“KRAS”) [21] were top ranked, that is, highly regulated, in Ago-IP_IgG_ (Supplementary Figure S1). These gene sets were also highly ranked in lysate (data not shown). As the Polycomb repressive complex 2 (PRC2) is known to transcriptionally silence targets by trimethylation of histones at H3 position K27, “H3K27me3 marked” and “PRC2 targets” are largely overlapping gene sets. PRC2 promotes oncogenic processes [22], while “KRAS” consists of genes downregulated in oncogenic *KRAS G12* mutant expressing prostatic epithelial cells [21].

To further prioritize *miR-375* targets for functional analysis, we selected genes being enriched at least 4-fold in the Ago-IP fractions and downregulated by a factor of at least 1.5 in the lysate of PC-3 *miR-375* compared to PC-3 control (Supplementary Figure S2). As an additional selection criterion, due to the technical variance of lowly expressed genes, the expression of target genes were required to be represented by at least 8 counts per million (cpm) in each experiment. This prioritization yielded 121 direct *miR-375* targets (Supplementary Table S4). The majority of these (88%) were protein coding genes. GO using Ingenuity pathway analysis (IPA) placed “cancer” (p = 0.0420 to 3.66 × 10^−6^), “cell cycle” (p = 0.0481 to 0.0013), “cell growth and proliferation” (p = 0.0434 to 0.0013), and “cell-to-cell signaling and interaction” (p = 0.0481 to 0.0013) among the most significantly associated GO categories. The gene coding for the MAP kinase signaling protein ERK, which regulates proliferation, migration and cell survival, was found to be the central node in the top network.

We next re-annotated our microarray data set (GSE29079), which consists of tumor (n = 47) and benign (n = 47) prostate tissue specimens showing differential *miR-375* expression [2], according to the method described in [23], to enable assignment of signals to newly annotated transcripts including long noncoding RNAs (using Ensembl v75 and the long noncoding RNA collection of Cabili et al. [24]). The scaling method Jetta [25] was subsequently used for differential expression analysis between tumor and benign samples. Sixty-three of the top 121 direct *miR-375* targets were downregulated in tumors overexpressing *miR-375* by a factor of 2.9 (p = 1.46 × 10^−16^; Supplementary Table S4). These 63 genes were most prominently described by the GO network “cell proliferation”, and cell cycle regulator *CCND1* was key upstream regulator (p = 0.0048). *CCND1* was also significantly upregulated in PC-3 *miR-375* compared to PC-3 control (1.4-fold, p = 0.03, Supplementary Table S2).

**Figure 3:**
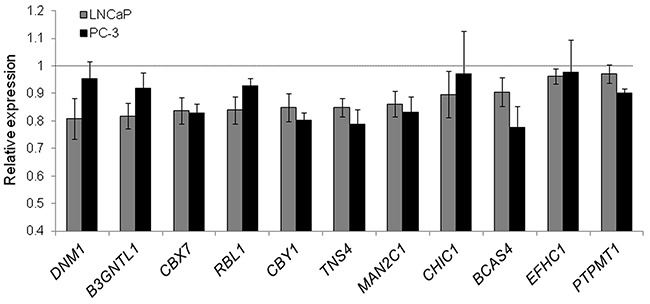
Validation of *miR-375* targets in LNCaP and PC-3 cells by qRT-PCR Relative expression of target RNAs in TL of LNCaP (grey) and PC-3 *miR-375* (black) cells normalized to TL of LNCaP or PC-3 control cells, respectively, after adjustment to *GAPDH*. All experiments were performed in three replicates. All error bars show s.d.. Significant difference in the expression of all target genes in LNCaP *miR-375*/PC-3 *miR-375* compared to LNCaP control/PC-3 control was observed (*P* ≤ 0.05), except for *DNM1*, *CHIC1* and *EFHC1* in PC-3 cells. Experiments were performed 48 h after transfection.

To validate these findings, we selected eleven genes (*B3GNTL1, BCAS4, CBX7, CBY1, CHIC1, DNM1, EFHC1, MAN2C1, PTPMT1, RBL1,* and *TNS4*), which are potential tumor-suppressors according to GO and a manual literature search and are predicted *miR-375* target genes [26]. We quantified them by qRT-PCR using TL fractions of PC-3 *miR-375* and PC-3 control cells as well as of LNCaP *miR-375* and LNCaP control cells 48 h after transfection and observed reduced mRNA expression levels following *miR-375* overexpression in all cases (Figure 3). Nine out of the eleven genes could be assigned to the pathway “cell cycle, cellular development, cancer” (Supplementary Figure S3).

### Potential *miR-375* targets are validated by *miR* sponge technology

**Figure 4:**
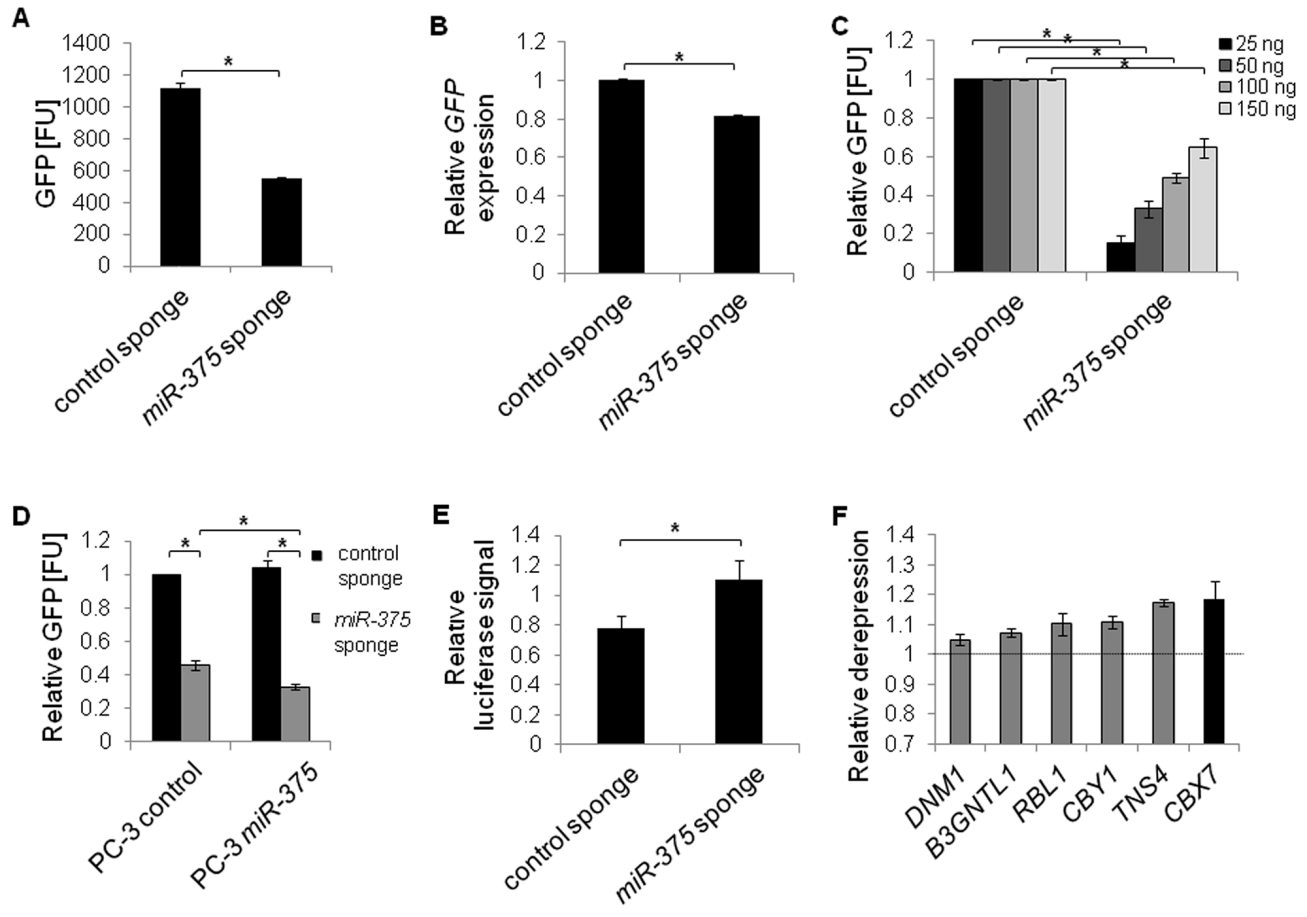
*MiR-375* target validation by miR sponges in PC-3 cells **A** and **B.** Stability of *miR-375* sponges was confirmed by measuring GFP protein (A) or mRNA expression (B), from pREP4-GFP sponge plasmids. FU = fluorescence units. **C** and **D.** Specificity of *miR-375* sponges. GFP protein levels (FU) from varying concentrations of sponge plasmids with endogenous *miR-375* (C), and following overexpression of *miR-375* (D). **E.** Functionality of *miR-375* sponges. Relative luciferase signal of pMirGlo-*miR-375*-target luciferase reporter following *miR-375* sponge or control sponge introduction, respectively, was adjusted to the Renilla signal and normalized to a non-targeting control. **F.** Derepression of *miR-375* targets following expression of *miR-375* sponge compared to control sponge. *GAPDH* served as internal control. All differences between *miR-375* sponge and control sponge in F were statistically significant (*P ≤ 0.05). All experiments were performed in three replicates. *P ≤ 0.05. All error bars show s.d. Measurements were performed 48 h after transfection.

MiR sponges have recently been introduced as molecular tools validating direct miR targets [27]. As the sponges decoy miRs and hence reduce the pool of endogenous miRs able to repress their targets, direct targets of miRs are de-repressed upon sponge introduction into cells. To validate the potential *miR-375* targets, we first tested the stability, specificity and functionality of *miR-375* sponges. Transfecting a pREP4-GFP reporter construct with ten *miR-375* binding sites in its 3′-UTR into PC-3 cells showed that sponge-mediated inhibition was favored over degradation: GFP protein was reduced by 51% (Figure 4A, Supplementary Figure S4), but GFP mRNA was decreased by only 18% (Figure 4B) 48 h after transfection. The *miR-375* sponges were specific, because translation was repressed by endogenous *miR-375* leading to lower GFP signal relative to the control sponge when sponge plasmids were expressed GFP at subsaturating levels 48 h after transfection (Figure 4C). The specificity of sponge repression by *miR-375* was further confirmed by sponge inhibition after ectopic *miR-375* expression 48 h after transfection (Figure 4D). To test the functionality of the sponges, we cloned an artificial *miR-375* target containing two complementary *miR-375* binding sites into a pMirGlo luciferase reporter vector and measured its de-repression upon *miR-375* sponge introduction after 48 h. The artificial target was de-repressed by the *miR-375* sponge (1.4-fold) compared to the control sponge demonstrating that the sponges were functional 48 h after transfection (Figure 4E).

After confirming stability, specificity and functionality of the *miR-375* sponges, we measured the expression of *miR-375* target genes in PC-3 cells transfected with either the *miR-375* or control sponge, focusing on the six *miR-375* target genes that showed the strongest downregulation in the qRT-PCR measurements in LNCaP *miR-375* cells (Figure 3). Ectopic expression of *miR-375* sponges in PC-3 cells resulted in upregulation of all six tested genes (*B3GNTL1, CBX7, CBY1, DNM1, RBL1, TNS4* Figure 4F) compared to the control sponge. We therefore defined these genes as the top validated *miR-375* targets. Among these, *CBX7* (chromobox homolog 7; Polycomb repressive complex member) exhibited the highest deregulation (1.2-fold). The observed degree of deregulation upon sponge induction is in accordance with the effects observed by Ebert et al. [27], who established the sponge technology.

### *MiR-375* enhances prostate cancer progression *in vitro* and *in vivo*

We hypothesized that *miR-375* might exert a tumor-promoting role in prostate cancer for the following reasons: 1) The newly identified *miR-375* targets were linked to tumor promoting networks, 2) *miR-375* expression levels are enriched in prostate cancer compared to normal tissue [2], and 3) serum and tissue levels correlate with prostate cancer progression [2, 7].

**Figure 5:**
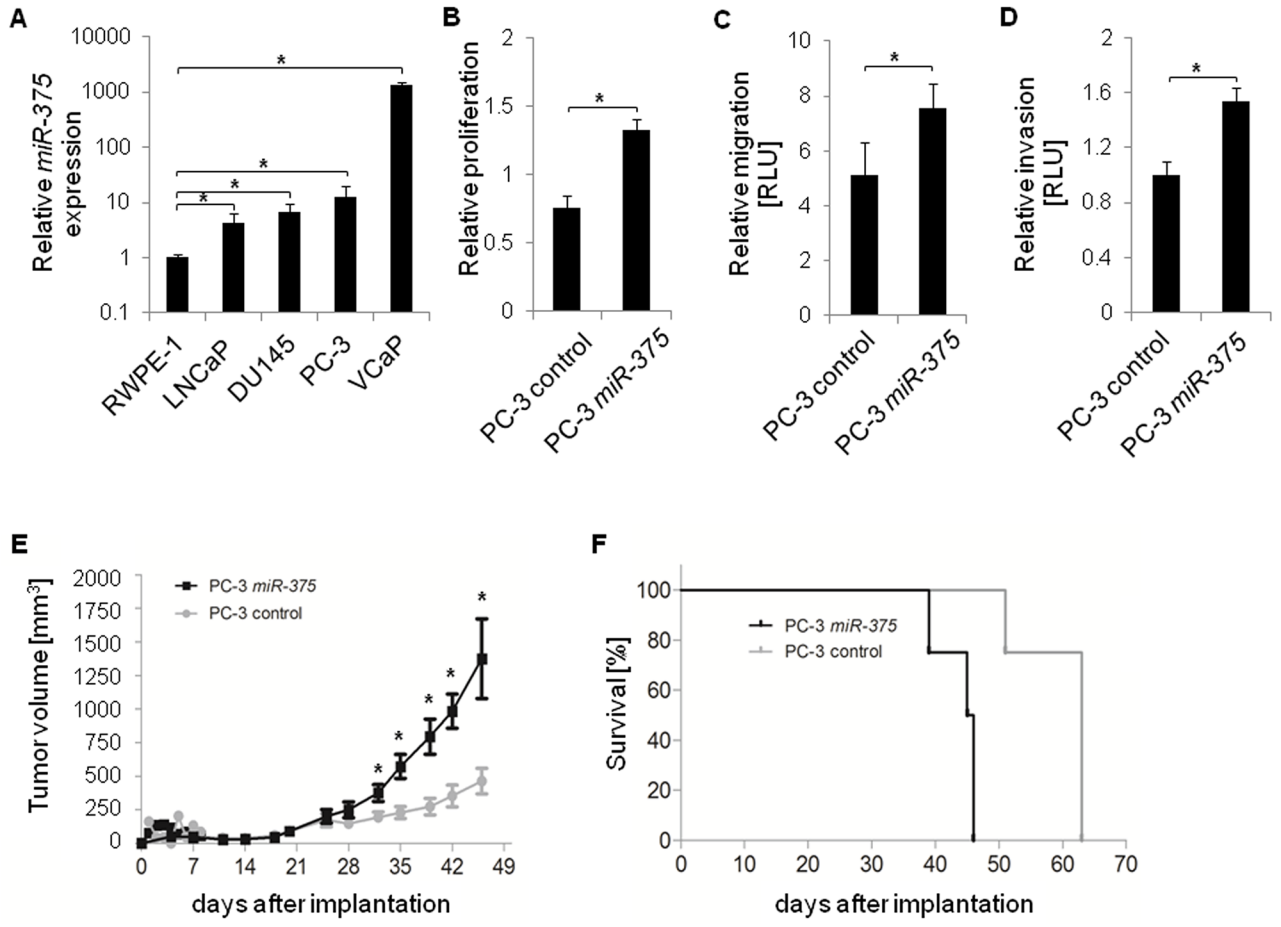
Functional analysis of *miR-375 in vitro* and *in vivo* **A.** Relative *miR-375* expression in various prostate cancer cell lines (LNCaP, DU145, PC-3, and VCaP) compared to control cells (RWPE-1). **B-D.** Relative proliferation (B), migration (C), and invasion (D) of PC-3 control cells and following *miR-375* overexpression. Proliferation, migration and invasion were measured 96 h, 48 h and 48 h after transfection, respectively. RLU = Relative Light Units. Experiments of A-D were performed in three replicates, with the exception of the invasion assay which was performed in four replicates. Error bars of A-D show s.d. *P ≤ 0.05. Proliferation, migration and invasion rates were measured 96 h after transfection. **E** and **F.** Tumor growth (E) and survival rate (F) in xenograft mice (n = 8 per subclone, total of 16 mice) following implantation of control PC-3 cells or PC-3 *miR-375* cells. Survival was defined as neither died nor having reached a tumor volume of 1500 mm^3^, where mice were sacrificed. Error bars of E show s.e.m. *P ≤ 0.05.

We first tested whether *miR-375* expression was elevated in prostate cancer cell lines LNCaP, DU145, PC-3, and VCaP (all derived from metastatic tissues) compared to the normal prostate cell line RWPE-1. *MiR-375* was significantly enriched in all tumor cell lines, LNCaP (4.1-fold, p = 1.69 × 10^−2^), DU145 (6.7-fold, p = 0.0031), PC-3 (12.4-fold, p = 0.0012) and VCaP (1341.8-fold, p = 1.34 × 10^−6^), compared to RWPE-1 cells (Figure 5A). Thus, the *in vitro* data were consistent with the elevated levels of *miR-375* in aggressive tumors [2]. To examine the effect of *miR-375* enrichment in prostate cancer cells, we performed cellular assays after transient *miR-375* overexpression. PC-3 and LNCaP cells overexpressing *miR-375* had a higher proliferation rate compared to controls (1.3-fold, p = 1.9 × 10^−6^, and 1.8-fold, p = 2.3 × 10^−7^, respectively, Figure 5B, Supplementary Figure S5A) as well as increased migration (1.5-fold, p = 0.05, Figure 5C) and invasion (1.5-fold, p = 8.9 × 10^−3^, Figure 5D) of PC-3 cells 96 h after transfection.

To examine whether tumorigenic processes are activated, we used the RNA-Seq data set obtained from TL of PC-3 *miR-375* and PC-3 control (Supplementary Table S2) to conduct GO analyses of genes upregulated (> 2-fold) in PC-3 *miR-375*. MAPK1 (p = 0.0089) and the PI3K complex (p = 0.0402) were the most highly activated upstream regulators, consistent with the network analysis identifying PI3K/AKT, MAPK, TGFB, and JNK as central signaling pathways of upregulated genes (data not shown). These are all known to play important roles in tumor cell proliferation, migration and invasion.

To test the oncogenic effect of *miR-375 in vivo*, PC-3 *miR-375* and PC-3 control cells were each injected into eight NMRI nu/nu mice, and tumor growth was monitored twice a week for 46 days. Thirty-two days after injection, the tumor volumes were significantly higher (p = 0.0148, 1.9-fold) in mice with PC-3 *miR-375* and were further increased to 3.0-fold at 46 days after injection (p = 0.0283, Figure 5E, Supplementary Figure S5B, C). On day 39, the survival rate of mice with PC-3 *miR-375* decreased to 75%, and none of these mice survived day 47 (Figure 5F). In contrast, all PC-3 control mice survived day 47, and a 75% survival rate was only reached 51 days after injection. The remaining PC-3 control mice died at day 63. Thus, survival of mice with PC-3 *miR-375* was significantly lower than those with PC-3 control (median survival rates 45.5 days and 63 days, respectively, p = 0.0001, Log-rank test). Survival was defined as neither died nor having reached a tumor volume of 1500 mm^3^, where mice were sacrificed.

Taken together, the oncogenic role of *miR-375* in prostate cancer was confirmed by GO analysis and functional assays, as *miR-375* enhanced proliferation, migration and invasion *in vitro*. Moreover, the xenograft model demonstrated that *miR-375* enhances tumor growth and progression and reduces survival *in vivo*.

### The *miR-375* target *CBX7* is associated with prostate cancer progression and tumor specific death

**Figure 6:**
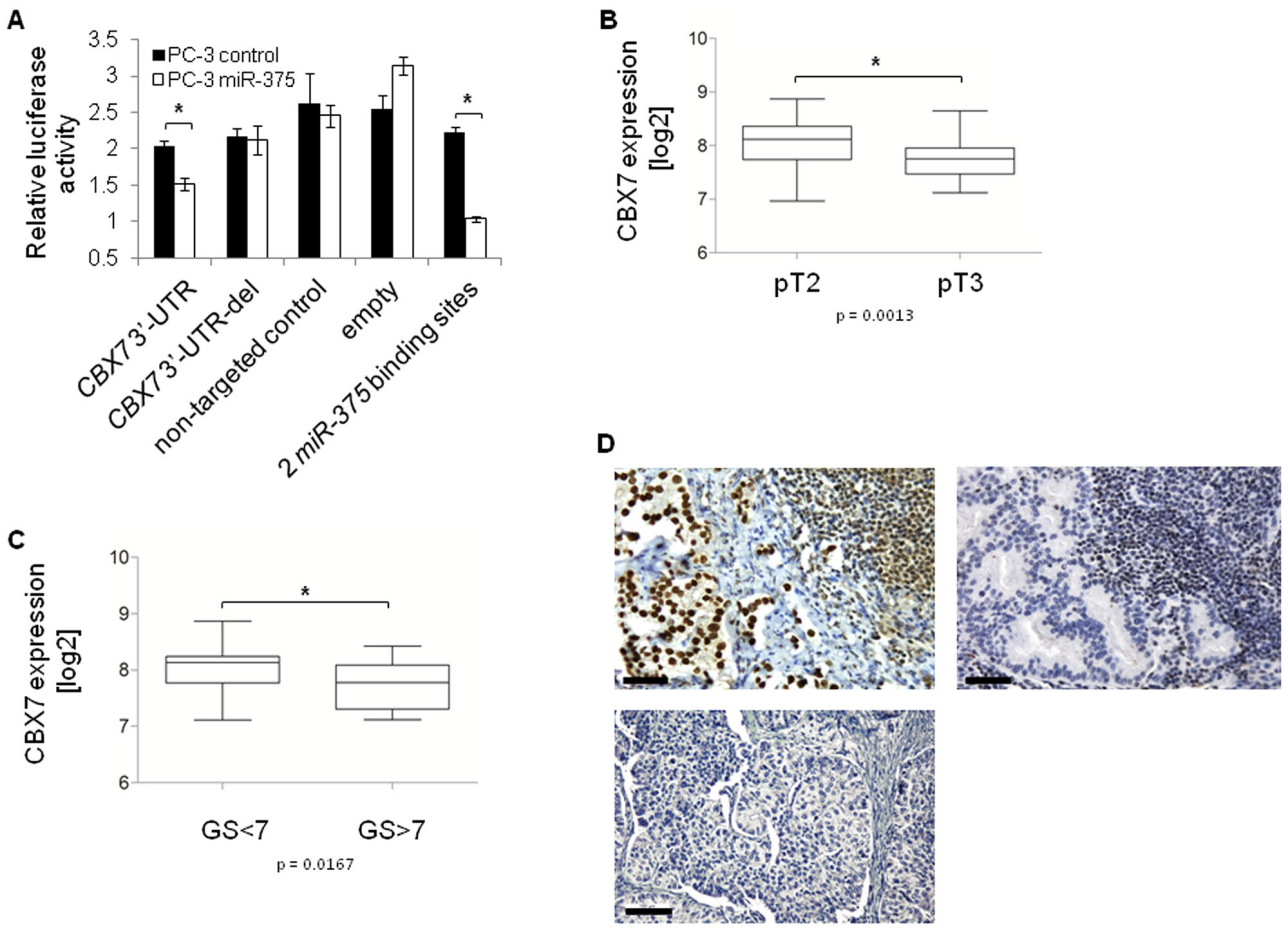
*CBX7* is a major target of *miR-375* in prostate cancer **A.** Luciferase signal assay confirms that *CBX7* is a direct target of *miR-375*. The experiment was performed in two replicates with quadruplicates. Error bars of A show s.d.. *P ≤ 0.05. The luciferase assay was performed 48 h after transfection. **B.** *CBX7* expression is decreased in pT3 (n = 38) compared to pT2 (n = 54) tissues, and **C.** lower in GS>7 (n = 12) compared to GS<7 (n = 27) tissues (GSE29079). Error bars of B and C show s.e.m. *P ≤ 0.05. **D.** Tissue microarray with CBX7 immunohistochemical staining of 58 lymph node metastases. CBX7 staining is positive in 17 [29.3%] (upper panel, left), but negative in 41 [70.7%] (upper panel, right) lymph node metastases. 20x magnification, scale bar = 50 μm. The lower panel shows the negative control (only secondary antibody). 10x magnification, scale bar = 100 μm.

To elucidate the oncogenic role of *miR-375* in prostate cancer on the cellular level, we focused on the *miR-375* target Polycomb repressive complex member *CBX7* because it was de-repressed the strongest of the top six validated target genes by *miR-375* sponges (factor 1.2, Figure 4F), and because GSA showed the involvement of *miR-375* in Polycomb regulation (Supplementary Figure S1). Finally, CBX7 is proposed to play an anti-oncogenic role as measured by proliferation [28], and invasion [29], and CBX7 loss results in highly malignant phenotypes and poor prognoses in other entities (colon, bladder, pancreas, breast, liver and lung cancer) [30–34]. Consistent with this, and with the strong downregulation in our data (to 71%; p = 7.6 × 10^−10^, GSE29079) as well as two validation prostate cancer tissue data sets (TCGA, Supplementary Figure S6A; Taylor et al., GSE21032), we hypothesized that the tumor-promoting role of *miR-375* might be explained by targeting *CBX7*. To confirm the direct targeting of CBX7 by *miR-375*, we cloned the 3′-UTR of *CBX7* into a luciferase reporter vector and measured the degree of signal reduction following *miR-375* overexpression in PC-3 cells after 48 h. The luciferase signal was significantly reduced by 26% (p = 0.0217, Figure 6A) in the *miR-375*-overexpressing cells, but not in those with the deletion control (lacking the *miR-375* binding sequence AUACGUGGGGUGGGUCUGGACAAGG), the non-targeting control or the empty vector. In contrast, the luciferase signal in cells expressing the positive control, an artificial *miR-375* target harboring two *miR-375* binding sites, was repressed following *miR-375* expression. Moreover, *in silico* analysis of the free binding energy [35] between *miR-375* and *CBX7* confirmed strong binding of *miR-375* to the 3′-UTR of *CBX7* (- 26.7 kcal/mol). The *miR-375* binding site is depicted in Supplementary Figure S6B.

As we hypothesized that *miR-375* mediated prostate cancer progression might be explained by its targeting of *CBX7*, we examined the associations between *CBX7* expression in prostate cancer tissue samples and tumor status and Gleason Scores (GSE29079). *CBX7* expression was significantly lower in advanced tumor stages (pT3 vs. pT2, p = 0.0013, Figure 6B) and in tumors with high (> 7) compared to low (< 7) Gleason Scores (p = 0.0167, Figure 6C). Dividing patients into subgroups with Gleason Score < 7 and > 7 was performed to clearly distinguish low risk from high risk patients.

Notably, *CBX7* expression was also decreased in pT3 vs. pT2 and Gleason Score > 7 vs. < 7 tumors in an independent data set published by Taylor et al. (GSE21032). Hence, *CBX7* was expressed inversely to *miR-375* in advanced tumors. In parallel, we investigated CBX7 expression in lymph node metastases from 58 prostate cancer patients by tissue microarray. We found that the majority of lymph node metastases (70.7%, n = 41) did not express CBX7 (Figure 6D). CBX7 absence in lymph node metastases was independent from the CBX7 status of the primary prostate cancer samples of the same patients (p = 0.530, Pearson Chi^2^ test, two-sided, Supplementary Table S5). These data were in accordance with the high expression of *miR-375* in lymph node metastasized prostate carcinoma [7]. In addition, CBX7 was not expressed in > 90% (11 of 12) of patients who had died of the tumor (median survival time: 40 months), but was not expressed in only 55% (11 of 20) of patients with no tumor specific death (median survival time: 75 months) (p = 0.03, Pearson Chi^2^ test, two-sided). Notably, the negative control (only secondary antibody) showed that the background was zero (Figure 6D).

Taken together, these data suggest that the oncogenic properties of *miR-375* might be explained by its targeting of CBX7, as *CBX7* expression was significantly decreased in primary tissues with high Gleason Scores and in those with advanced tumor stages. Furthermore, CBX7 absence was also associated with lymph node metastases and prostate cancer specific death.

### Repression of *CBX7* by *miR-375* leads to upregulation of *CBX8* and activation of transcriptional programs associated with malignant progression

**Figure 7:**
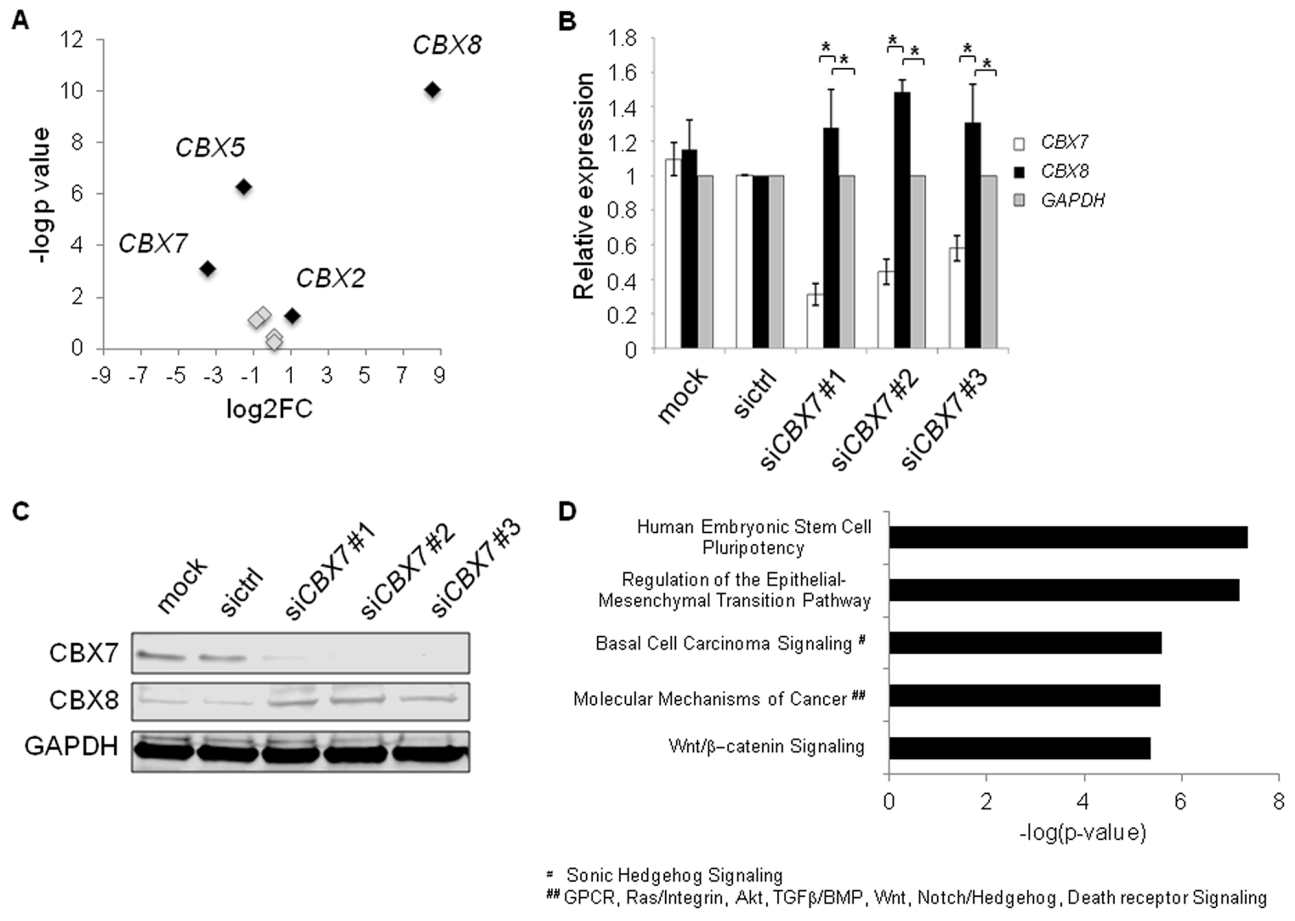
**A.** Expression of CBX genes in RNA-Seq data of *miR-375* overexpressing PC-3 cells. *MiR-375* regulated *CBX* genes (> 2-fold) are highlighted in black, whereas non-regulated *CBX* genes are depicted in grey. **B** and **C.** Knockdown using si*CBX7* leads to enforced *CBX8* expression in LNCaP cells on RNA (B), and protein (C) levels compared to mock or sictrl transfection. GAPDH was used as control. **D.** Top activated pathways of CBX7 targets following *CBX7* knockdown in LNCaP cells as identified by microarray analysis. Experiments were performed in triplicates. *P ≤ 0.05. Error bars show s.d. Experiments were performed 48 h after transfection.

CBX2, 7, and 8 are mutually exchangeable subunits of PRC1 [36]. Loss of CBX7 leads to higher abundances of CBX2 and CBX8, and preferential incorporation of CBX8 (and to a lesser extent CBX2) into PRC1 [36]. Therefore, we hypothesized that *miR-375* might regulate the sensitive and dynamic equilibrium of CBX7 and CBX8 expression, which is known to impact transcriptional programs in development and cancer [36–38]. To this end, we investigated the expression of *CBX* genes in PC-3 cells overexpressing *miR-375*. *CBX2* and *CBX8* were upregulated in our RNA-Seq data of PC-3 *miR-375* by 2.1-fold and 365-fold, respectively 48 h after transfection (Figure 7A, Supplementary Table S2). Furthermore, there was a clear inverse relationship between *CBX7* and *CBX8* (and to a minor extent *CBX2*) mRNA expression levels of tumor and normal samples in the TCGA tumor data set overexpressing *miR-375* (Supplementary Figure S6A). The Pearson correlation analyses of *miR-375/CBX7*, *CBX7/CBX8,* and *CBX7/CBX2* showed that *CBX7* was significantly negative co-expressed to *miR-375* (r = - 0.58, p < 0.0001, Supplementary Figure S6C), *CBX8* (r = - 0.33, p < 0.0001, Supplementary Figure S6D), and *CBX2* (r = - 0.34, p < 0.0001; Supplementary Figure S6E). These data were further corroborated in the data set published by Taylor et al. (GSE21032) showing significant downregulation of *CBX7* (p = 7.00 × 10^−13^), and upregulation of *CBX8* (p = 0.0031) as well as *miR-375* (p = 6.36 × 10^−7^, data not shown). To investigate whether CBX7 loss leads to upregulation of CBX8 in prostate cancer cells, we transfected LNCaP cells with siRNAs against *CBX7*, thereby emulating the effect of *miR-375* de-repression. *CBX7* knockdown was confirmed by both qRT-PCR analysis and western blot 48 h after transfection (Figure 7B, 7C). As expected, all three *CBX7* siRNAs resulted in loss of CBX7 and significantly increased levels of *CBX8* mRNA (Figure 7B) and protein (Figure 7C). Notably, even small changes in *CBX7* and *CBX8* expression affect the sensitive and dynamic equilibrium of CBX7 and CBX8 [36, 37]. We concluded that *miR-375*-mediated *CBX7* repression upregulates CBX8, thereby influencing the critical levels of CBX7 and CBX8 in PRC1 needed for distinct transcriptional programs.

We next analyzed the molecular signatures following CBX7 silencing in prostate cancer cells after 48 h using global microarray analysis. We integrated known CBX7 targets [36, 37] with genes upregulated following *CBX7* knockdown in LNCaP cells from the microarray analysis (Supplementary Table S6, E-MTAB-3730) and found activation of prominent cancer pathways such as epithelial to mesenchymal transition (EMT) and Wnt/β-catenin signaling pathways (Figure 7D). We also integrated known CBX7-regulated genes with genes overexpressed upon *miR-375* in the RNA-Seq data set (Supplementary Table S2), and found the same activated pathways (Supplementary Figure S7), indicating that the activation of these pathways by *CBX7* knockdown is mediated by *miR-375* in prostate cancer cells. Taken together, these data suggest that *miR-375* leads to the activation of oncogenic signatures and tumor progression by targeting *CBX7.*

## DISCUSSION

The aim of this study was to characterize the promising prostate cancer progression marker *miR-*375. MiRs are assumed to regulate the majority of transcripts within a cell, and hence deregulation of miRs is frequently implicated in diseases including cancer. A key for understanding the role of a specific miR in tumorigenesis is the identification of its target genes. Several crosslinking-based, immunoprecipitation methods to profile miR:target binding have been developed in recent years [39, 40]. These genome-wide analyses have shown that canonical targeting rules like miR binding to the 3′-UTR of a target RNA or seed complementarity might apply to only a fraction of miR:mRNA interactions [15] and may thus explain the low accuracy of commonly used miR target prediction programs. Our Ago-RIP-Seq approach after miR overexpression was designed to overcome this limitation. It is also more precise than transcriptomic profiling after miR overexpression alone as the latter cannot dissect primary from secondary targets or indirect target modulation. Moreover, compared to the array-based Ago-RIP-Chip technology [18], Ago-RIP-Seq is more sensitive, does not rely on prior annotation of transcripts, and enables the identification of all RNA classes, including noncoding and antisense RNAs. This is important, as recent studies have shown that noncoding RNAs can be targeted by miRs and thereby affect tumorigenesis [41]. In addition, our vector-based overexpression of the miR at physiological concentrations overcomes the limitations of approaches relying on mimic miRs [16], where the degree of incorporation into functional Ago complexes as well as the extent of off-target effects due to supra-physical miR levels are unknown [42]. MiR overexpression in the physiological range can be assumed when the resulting miR levels do not exceed the abundance of the most highly expressed miR in the cell. In PC-3 cells, endogenous *miR-99a* was expressed 6-fold higher than overexpressed *miR-375* (data not shown). Hence, we considered the overexpression as physiological. To correct for unspecific IP binding as well as secondary transcriptomic changes, that contribute to the high intrinsic noise of IP experiments [17, 43], we established a new experimental approach and sequenced total lysates as well as control isotype IgG-IP fractions of both miR overexpressing and control cells. We also devised a novel universal data analysis strategy for this two-factorial setup.

To prioritize novel potential *miR-375* targets for functional characterization, we selected targets enriched in Ago-RIP fractions and decreased in total lysate fractions following *miR-375* overexpression, and integrated tissue gene expression data, GSA and GO analysis, as well as literature searches. These analyses indicated a role of *miR-375* in oncogenic processes via the regulation of genes involved in Polycomb complex mediated transcriptional control. In particular, Polycomb target genes carrying H3K27me3 marks were highly regulated by *miR-375*. We identified the upstream regulator and Polycomb group member *CBX7* as a major target of *miR-375*. *CBX7* encodes a PRC1 subunit, which is involved in gene repression by guiding PRC1 to PRC2-methylated (H3K27me3) promoters [44]. We reasoned that the widespread oncogenic gene expression changes mediated by *miR-375* may be explained by its ability to repress *CBX7*. In support of this hypothesis, we observed strong *CBX7* depletion in TL of *miR-375* cells, high enrichment in Ago-IP fractions, and the highest de-repression of the top six validated targets following *miR-375* sponge expression. *In silico* analysis and luciferase reporter assays in PC-3 cells confirmed the direct binding of *miR-375* to the 3′UTR of *CBX7* RNA. *CBX7* expression was significantly decreased in tumors with a high Gleason Score, and in those, which extended through the prostate capsule, and hence showed an inverse expression pattern to *miR-375*. These observations were in concordance with the enhanced migration and invasion of *miR-375* overexpressing prostate cancer cells. In contrast, CBX7 decreased invasion [29] and is proposed to be involved in cellular processes implicated in migration [45]. Moreover, we observed CBX7 loss in lymph node metastases and showed that this correlates with prostate cancer specific death. These findings are in agreement with other studies in colon [30], bladder [31], pancreatic [32], breast [33], gastric [46], thyroid [47], liver and lung [34] cancers, in which the loss of CBX7 is correlated with a highly malignant phenotype and the retention of CBX7 expression with a longer survival of colon [30] and pancreatic cancer patients [32]. The tumor-suppressing role of CBX7 has also recently been shown in Cbx7 knockout mice [34]. In lung cancer, CBX7 restoration resulted in decreased proliferation and increased apoptosis [48]. Moreover, in breast cancer, low expression of CBX7 may serve as prognostic marker [49]. Of note, these findings are in contrast to a study where an oncogenic role for CBX7 was suggested [50]. However, this finding was based on the observation of notable growth arrest in CBX7 knockdown LNCaP cells, which could neither be seen in other prostate cancer cell lines nor after CBX7 overexpression in LNCaP cells [45].

*MiR-375*-mediated repression of *CBX7* leads to higher levels of CBX8 in prostate cancer cells. This was supported by downregulation of *CBX7* and upregulation of *CBX8* in *miR-375* overexpressing prostate tissues. PRC1 containing either CBX7 or CBX8 is well known to modulate different developmental processes: For example, the exchange of CBX7 to CBX8 induces distinct transcriptional programs and differentiation processes in hematopoetic and embryonic stem cells [36, 37]. The balance of CBX7 and CBX8 expression is also known to play an important role in glioblastoma where CBX7 is depleted and CBX8 is abundantly expressed [51]. In agreement with this, other studies on colon [52], esophageal [53] and breast cancer [54] found that PRC1 containing CBX8 mediates oncogenic properties. Notably, a recent study in prostate cancer indicated that CBX2- and CBX8-containing PRC1 promotes the progression of prostate cancer to a highly aggressive neuroendocrine tumor subtype [55]. To investigate the molecular signatures induced by CBX7 repression in prostate cancer cells, we performed expression profiling following siCBX7 knockdown and integrated our data with known CBX7 targets [36, 37]. We found that pathways commonly deregulated in cancer such as the EMT and Wnt/β-catenin signaling pathways were activated. CBX7 is capable of upregulating E-cadherin [56] indicating that it plays a critical role in later stages of cancer progression [47]. These data are also in line with a previously published study showing that CBX7 inhibits tumor progression by repression of the Wnt/β-catenin pathway in breast cancer [57]. When we compared the expression profiles of gene upregulated following *miR-375* overexpression with the known CBX7 targets, we found the same pathways, indicating that the activation of these oncogenic signatures by CBX7 repression is influenced by *miR-375*. Hence, we reason that *miR-375* exerts its oncogenic properties by targeting CBX7 and thus regulating important cancer pathways. This is in agreement with the increased expression of *miR-375* in prostate cancer compared to normal tissues as well as in the prostate cancer cells DU-145, VCaP, LNCaP and PC-3 compared to benign RWPE-1 cells. It is also supported by our finding that overexpression of *miR-375* leads to enhancement of proliferation, migration and invasion *in vitro*, induction of tumor growth and progression as well as reduced survival *in vivo*.

Our data suggest that targeting the Polycomb complex regulated epigenome might be a reasonable strategy to inhibit prostate cancer progression [58]. This could be achieved by either employing the tumor suppressive role of CBX7, as it has been proposed for breast cancer [57], or by controlling *miR-375* levels. To this end, it has been shown that a miR-based therapy is promising in an *in vivo* preclinical prostate cancer model (reviewed in [59]). *MiR-375* sponges, which we have shown to inhibit *miR-375* and to de-repress *CBX7 in vitro*, may potentially be applied *in vivo*. Recent reports have provided encouraging data for the successful application of the miR sponge technology to inhibit oncomiRs *in vivo,* thereby preventing metastasis formation [60].

## MATERIALS AND METHODS

### Cell lines

The cell lines LNCaP (CRL-1740) and PC-3 (CRL-1435) were purchased from ATCC between 2012 and 2013, and cultured according to manufacturer's instructions. Their identity was verified by SNP profiling conducted at Multiplexion GmbH.

### Xenograft model

This xenograft study was carried out in accordance with the recommendations in the Guide for the Care and Use of Laboratory Animals of the Society of Laboratory Animals (GV SOLAS). All animal experiments were approved by the Committee on the Ethics of Animal Experiments of the regional council (Regierungspräsidium Freiburg, Permit Number: G-13/13). Xenografts were established by subcutaneous injection of 5 × 10^6^ PC-3 *miR-375* or PC-3 control cells into the left flank of male NMRI nu/nu mice, respectively (n = 8 per subclone, total of 16 mice; Harlan, Denmark). Tumor growth and body weight were recorded twice a week for 46 days. Tumor growth was followed by serial caliper measurement. Tumor volumes were calculated by using the formula (a × b2)/2, where length (a) was the largest dimension and width (b) the smallest dimension perpendicular to the length. When mice had not died yet, and individual tumors reached a volume of 1500 mm^3^ the mice were sacrificed and tumors were harvested. Values are prepresented as mean ±SEM.

### *Pre-miR-375* cloning

For *miR-375* overexpression, *pre-miR-375* was amplified from a DNA pool containing equal amounts of LNCaP, PC-3, H1299, DU145, A549, H1650 and H1975 DNA with the primers *pre*-*miR-375*_*Kpn*I_F1 and *pre*-*miR-375*_*Bam*HI_R1 listed in Supplementary Table S7. Amplicons were cloned into the *Kpn*I/*BamH*I-linearized episomal expression vector pREP4 (Invitrogen) generating the pREP4_*miR-375* vector. The pre-miR:vector clone was confirmed by Sanger sequencing (GATC Biotech AG, Konstanz, Germany) using pREP fwd, pREP rev and EBV reverse primer, respectively (Supplementary Table S7).

### *MiR-375* sponge generation

*MiR-375* sponge cloning was performed using a single directional ligation into the nonpalindromic *SanD*I site of the pREP4 vector as previously described [61]. As the episomal expression vector pREP4 harboring GFP contains multiple *SanD*I sites in the vector backbone, we first used the pcDNA3.1(+) vector for sponge subcloning after introducing a *SanD*I site into pcDNA3.1(+) using a linker and the *Nhe*I and *Xho*I sites. Vector:miR binding site duplex ligation using a ratio of 1:1000 generated clones with various numbers of *miR-375* binding sites. The clones with ten *miR-375* binding sites (which has been suggested to be optimal for sponge function [61]) were used for consecutive subcloning into the 3′-UTR of GFP within the pREP4 expression vector via the *Nhe*I and *Xho*I sites. As a negative control, we used the non-miR-target sequence described by Ebert et al. [27] for cloning into the 3′-UTR of GFP within the pREP4 expression vector. Linker sequence and miR binding site duplexes are provided in Table S7. Correct sponge cloning was verified by Sanger sequencing (GATC; Supplementary Table S7).

### Ago immunoprecipitation (Ago-IP)

Ago-IP was performed as previously described [17] with the following modifications: cell lysates were incubated with Protein G Sepharose beads (GE Healthcare Europe GmbH) 48 h after transfection for preclearing. Anti-pan-Ago antibody 2A8 (10 μg, Abcam) or IgG1 isotype control G3A1 (Cell Signaling) were coupled to the beads; and IP was performed overnight rotating at 4 °C. Ago-IPs were performed in three independent biological replicates.

### Library generation

cDNA libraries were generated from RNAs of the TL, Ago and IgG IP fractions by using the SMARTer Stranded Total RNA Sample Prep Kit (Takara Clontech). The samples (50 bp) were paired-end sequenced on an Illumina HiSeq 2000 sequencer. We obtained between 18.9 and 28.5 (median 23.7) million reads (Supplementary Table S7). Detailed information is given in the Supplementary Material and Methods.

### Bioinformatic data analysis

Paired-end sequencing reads were mapped to the human reference genome (hg19) using STAR aligner (version 2.3.1z4). Read counts were calculated by HTSeq-count (version 0.6.0.) Parameter details are provided in the Supplementary Material and Methods.

### Statistical modeling and analysis of differential Ago-RIP-Seq and RNA-Seq data sets

We normalized the Ago-RIP-Seq and RNA-Seq count data using the Trimmed Mean of M values “TMM” approach [62] within the edgeR package, as recommended for these types of experiments [63, 64], and fitted a negative binomial model to the read counts using the statistical pipeline of edgeR, R version 3.0.2. The negative binomial model is a widely used approach for RNA-Seq or related (e.g. RIP-Seq [65]) data. In parallel, we fitted a GLM to the resulting experimental design. The identification of direct *miR-375* targets was achieved by using the concept of linear contrasts, that is, linear combinations of the factor-levels:

Following *miR-375* overexpression, the fraction of *miR-375* and its targets is increased in the Ago complex. As there are still many other miR:target pairs associated with Ago, adjustments of the Ago-IP of PC-3 *miR-375* to the Ago-IP of PC-3 control was required to result in highly enriched *miR-375*:target pairs. To further reduce the noise in the RIP-Seq experiment [43] due to unspecific RNA binding to sepharose beads, the Ago-IP fractions were adjusted to IgG-IP (Ago-IP_IgG_) before comparing the Ago-IP of PC-3 *miR-375* with the Ago-IP of PC-3 control as follows:

$$(Ago.miR\text{-}375\;-\;IgG.miR\text{-}375\text{)}-(Ago.control\;-\;IgG.control\text{) [}Ago\text{-}IP_{IgG}\text{]}$$

In addition, miR overexpression leads to widespread secondary changes within the gene expression profiles, which might impact the IP profiles [17]. Thus, it was also important to adjust the levels of RNAs detected in the Ago-IP fractions to their expression levels measured in the total lysates (Ago-IP_TL_) as follows:

$$(Ago.miR\text{-375}-TL.miR\text{-}375\text{)}-(Ago.control-TL.control)\text{ [}Ago\text{-}IP_{TL}\text{]}$$

Moreover, as miR targets are decreased after miR expression, we also defined the comparison lysate for identifying whole transcriptomal changes, as follows:

(TL.miR-375−TL.control) [lysate]

The contrasts Ago-IP_IgG_, Ago-IP_TL_ and lysate define the type of comparison between factor levels. Their corresponding regression coefficients due to linear model fitting reflect the estimated fold change value (on log2 scale). Likelihood-ratio tests were applied for testing the regression coefficient on zero. Resulting p-values are Benjamini-Hochberg adjusted. Genes with an average count per million less than 1 were excluded from the analysis.

### Statistical analysis

If not stated otherwise, two-sided unpaired t-tests were performed to determine whether there were significant differences between treatments and their corresponding controls. A P ≤ 0.05 was indication of a statistically significant difference. All values presented as the mean±SD.

### Accession numbers

Sequencing data and microarray data are deposited under ArrayExpress accession numbers E-MTAB-3691 and E-MTAB-3730, respectively.

## SUPPLEMENTARY MATERIALS AND METHODS, FIGURES AND TABLES












