# Supplementary material for: Ago-RIP-Seq identifies Polycomb repressive complex I member CBX7 as a major target of miR-375 in prostate cancer progression
Source: Oncotarget. 2016 Jul 20;7(37):59589–603. doi: 10.18632/oncotarget.10729 (PMC5312160; doi:10.18632/oncotarget.10729)
Supplement: Supplementary file 1 [file oncotarget-07-59589-s001.pdf]

Ago-RIP-Seq identifies Polycomb repressive complex I member CBX7 as a major target of *miR-375* in prostate cancer progression

SUPPLEMENTARY MATERIALS AND METHODS

Cell culture and transfection

The cell lines LNCaP (CRL-1740) and PC-3 (CRL-1435) were purchased from ATCC and cultured according to manufacturer's instructions. For *miR-375* overexpression, PC-3 and LNCaP cells were transfected with pREP4_pre-*miR-375* vector using X-treme Gene HP transfection reagent (Roche) according to the manufacturer's instructions to generate PC-3 *miR-375* and LNCaP *miR-375* cells, respectively. Cells transfected with empty pREP4 vector were transfected accordingly (PC-3 control and LNCaP control, respectively). For *CBX7* knockdown, LNCaP cells were transfected with siRNAs against *CBX7* using X-treme Lipofectamine RNAi max (Invitrogen) according to the manufacturer's instructions. SiRNAs CBX7_1 (SI03128489), CBX7_2 (SI03189137), CBX7_3 (SI04270560), and sictrl (SI03650318) were purchased from Qiagen (sequences are listed in Tab. S7). For downstream applications, cultured cells were lysed in PLB buffer [1] for 5 min on ice and frozen overnight at −80°C 48 h after transfection.

Library generation

Purified RNAs of the TL, Ago and IgG IP fractions were quantified using a NanoDrop spectrometer and the quality was assessed by Agilent capillary electrophoresis using RNA picoChips. 10 ng of RNA were *DNase*I (Qiagen) treated, and rRNA was depleted by RiboZero (Epicentre). Sequencing libraries were generated with the SMARTer Stranded Total RNA Sample Prep Kit (Takara Clontech). Enrichment and size distribution of the libraries were quality-assessed by capillary electrophoresis on a DNA high sensitivity chip (Agilent). For sequencing, all six samples of one replicate of an IP experiment (TL, Ago-IP, and IgG-IP fractions of both PC-3 *miR-375* and PC-3 control) were pooled to generate a final concentration of 5 nM per lane. Three biological replicates were analyzed. The samples (50 bp) were paired-end sequenced on an Illumina HiSeq 2000 sequencer. Raw data were sorted and analyzed according to barcoded library adaptors without allowing a mismatch within the barcode sequence.

Bioinformatic data analysis

Paired-end sequencing reads were mapped to the human reference genome (hg19) using STAR aligner (version 2.3.1z4) with default settings except for the following parameters: 1) adaptor clipping, (--clip3pAdapterSeq TCGTATGCCGTCTTCTGCTTG), 2) clipping of the first 6 bases from both mates due to low sequence complexity (--clip5pNbases 6); and 3) permission to map to maximum 5 locations to decrease the amount of multimappers, that is, reads mapped to multiple locations (--outFilterMultimapNmax 5). Read counts were calculated by HTSeq-count (version 0.6.0) with default settings ("stranded" and "union") and Gencode annotation (version 17).

Quantitative real time polymerase chain reaction (qRT-PCR)

To quantify miR and mRNA, total RNA was first isolated using the miRNeasy kit (Qiagen) with additional *DNase*I (Qiagen) treatment. mRNA was reverse transcribed using the RevertAid H Minus First Strand cDNA Synthesis kit and random primer (both Thermo Scientific) according to manufacturer's instructions. Amplification and quantification of cDNA equivalent to 10 ng RNA was performed by adding a mixture of 5.5 µl Probes 480 Master Enzyme Mastermix (Roche), 0.11 µl of 20 µM primer pairs, 0.11 µl Universal Probe Library probe (UPL, Roche), and 0.28 µl H_2O on a LightCycler 480 PCR system (Roche). Cp values were adjusted to the housekeeping gene *GAPDH* and relative expression was calculated according to Livak and Schmittgen [2]. qRT-PCR primers are listed in Table S7. MiR expression levels were accessed using TaqMan MicroRNA Assays (Applied Biosystems).

Western blotting

PC-3 or LNCaP cells were lysed 48 h after transfection with either vector or siRNA with PLB buffer [1]. Cell debris was removed by centrifugation. Protein concentrations were measured by NanoDrop. Lysates were separated onto a denaturating polyacrylamide gel and the proteins were blotted onto a PVDF membrane. Reagents and devices were used from the Trans-Blot Turbo System (Biorad) were used according to the manufacturer's protocol. Ago, GAPDH, CBX7 and CBX8 proteins were detected by monoclonal mouse anti-pan-Ago 2A8 (1:1000, Abcam), monoclonal rabbit anti-GAPDH14C10 (1:1000, Cell Signaling), polyclonal rabbit anti-CBX7 ab21873 (1:1000, Abcam) and anti-CBX8 ab09031 (1:500,

Millipore) antibodies, respectively. Signals were detected with HRP-linked secondary antibodies (Pierce) using the Odyssey Imaging System (LI-COR Biosciences).

Proliferation assays

Proliferation of PC-3 and LNCaP cells transfected with either pREP4_pre-*miR-375* (PC-3/LNCaP *miR-375*) or empty vector (LNCaP/PC-3 control) was measured using the Wst1 assay (Roche) according to the manufacturer's protocol. Absorbance was quantified on a TECAN Infinite 200 reader (Tecan) 96 h after transfection and adjusted to absorbance detected at 24 h after transfection. Assays were performed in three biological replicates.

Transwell migration and invasion assays

Migration and invasion were monitored for 48 h using 6.5 mm transwell polycarbonate membrane inserts (8.0 mm pore size) in a 24-well plate (Greiner Bio One). Cell invasion was examined using Matrigel (BD Biosciences). 2.2 x 10^4 cells were resuspended in 200 µl serum-free medium and seeded on top of the membranes either coated with Matrigel or without. 750 µl medium containing 10% FCS were added to the lower well as attractant. 48 h after seeding, cells that had not migrated or invaded, respectively, were collected from the upper well. For quantification of migration or invasion, cells on the bottom of the insert and in the lower chamber were trypsinized and quantified using the Celltiter Glo luminescent cell assay (Promega) according to the manufacturer's protocol. Oxyluciferin emission of ATP-producing cells was detected using a TECAN Infinite 200 reader (Tecan). Wells containing medium without cells were used as a background control. Relative amount of migrated or invaded PC-3 *miR-375* cells were adjusted to PC-3 control cells. Assays were performed with at least three biological replicates.

Luciferase reporter assay

For luciferase sensor assays, 2 × 10^4 PC-3 cells were co-transfected with pMirGlo (Invitrogen) either containing the 3'-UTR of *CBX7*, the 3'-UTR of *CBX7* without the *miR-375* binding site (deletion control), miR non-targeted control, two complementary *miR-375* binding sites, or empty vector together with either pREP4_pre-*miR-375* or pREP4 control vector using X-treme Gene HP transfection reagent (Roche). Cells were lysed 48 h after transfection. Luciferase and Renilla signals were measured using the Dual-Glo Luciferase Assay System (Promega) according to the manufacturer's protocol. Firefly signals were normalized to the Renilla signals. Assays were performed in three technical and two biological replicates.

Gene ontology (GO) analysis

Data were analyzed using the QIAGEN's Ingenuity® Pathway Analysis (IPA®, QIAGEN Redwood City, www.qiagen.com/ingenuity).

Microarray analysis

RNA isolation and analysis

Total RNA of three biological replicates of LNCaP cells transfected with siCBX7_1 (SI03128489), siCBX7_2 (SI03189137), siCBX7_3 (SI04155312), or sictrl (SI03650318) (all purchased from Qiagen, Supplementary Table. S7) was isolated using the Qiagen miRNeasy kit according to the manufacturer's instructions. The quality of total RNA was checked by gel analysis using the total RNA Nano chip assay on an Agilent 2100 Bioanalyzer (Agilent). All samples had RNA index values greater than 9.

Probe labeling and illumina sentrix beadChip array hybridization

This work was performed in the Genomics and Proteomics Core Facility at the German Cancer Research Center (DKFZ), Heidelberg, Germany. Biotin-labeled cRNA samples for hybridization on Illumina Human Sentrix-12 BeadChip arrays (Illumina,) were prepared according to Illumina's recommended sample labeling procedure based on the modified Eberwine protocol [3]. In brief, 200 ng total RNA was used for complementary DNA (cDNA) synthesis, followed by an amplification/labeling step (*in vitro* transcription) to synthesize biotin-labeled cRNA according to the Illumina® Total Prep™ RNA Amplification Kit (Life Technologies). Biotin-16-UTP was purchased from Roche Applied Science. The cRNA was column purified according to the TotalPrep RNA Amplification Kit, and eluted in 60 µl of water. Quality of cRNA was controlled using the RNA Nano Chip Assay on an Agilent 2100 Bioanalyzer and spectrophotometrically quantified (NanoDrop).

Hybridization was performed at 58°C in GEX-HCB buffer (Illumina) at a concentration of 100 ng cRNA/µl, unsealed in a wet chamber for 20 h. Spike-in controls for low, medium and highly abundant RNAs, as well as mismatch control and biotinylation control oligonucleotides, were added. Microarrays were washed once in High Temp Wash buffer (Illumina) at 55 °C and then twice in E1BC buffer (Illumina) at room temperature for 5 min (washed in between with ethanol at room temperature). After blocking for 5 min in 4 ml of 1% (wt/vol) Blocker Casein in Hammarsten grade phosphate buffered saline (Pierce Biotechnology), array signals were developed by a 10 min incubation in 2 ml of 1 µg/ml Cy3-streptavidin (Amersham Biosciences) solution and 1% blocking solution. After a final wash in E1BC, the arrays were dried and scanned.

Scanning and data analysis

Microarray scanning was conducted using an iScan array scanner. Data extraction was carried out for all beads individually, and outliers were removed when < 2.5 MAD (median absolute deviation). All remaining data points were used for the calculation of the mean average signal for a given probe, and standard deviation for each probe was calculated. Raw signals were quantile normalized.

Immunohistochemistry

A prostate tissue microarray (TMA) containing prostatectomy specimens from a total of 164 patients was used for CBX7 staining. The TMA was constructed by the Tissue Bank of the National Center for Tumor Diseases Heidelberg (Ethics vote of the Medical Faculty Heidelberg 206/2005, 207/2005). All patients underwent radical prostatectomy between 1990 and 2010. A total of 578 prostate cancer tissue cores were analyzed, with each patient tumor represented by a median of 4 cores (range 1-5). Additionally, 35.4% (58 out of 164 patients) cores from lymph node tissue containing prostate cancer metastases were available for staining and analysis. Prostate cancer specimens were stained using antibodies against CBX7 (1:100 in PBS HPA 056480 rabbit pAb, Atlas). TMA slides were deparaffinized with xylene and rehydrated in a graded ethanol series. Antigen recovery was performed by heat retrieval using the Antigen Retrieval Solution (Dako). Blocking and immunodetection were performed using the HistoStain Plus kit (Life Technologies). Sections were counterstained with hematoxylin (Thermo Scientific), dehydrated in a graded ethanol series and mounted using Histomount media (Life Technologies). TMA cores were analyzed based on the intensity of the staining and percentage of positive cells. Values between 1 and 12 were were used to score CBX7 expression. The threshold for positivity was set at 6, and negative cores were assigned a value of 0. Analyses were conducted using the SPSS version 17.0 software (IBM, DE, USA). To evaluate correlations of clinic-pathological data and staining results, a two-sided Pearson Chi square test was performed. P values < 0.05 were considered significant.

REFERENCES

1. Tan LP, Seinen E, Duns G, de Jong D, Sibon OC, Poppema S, Kroesen BJ, Kok K and van den Berg A. A high throughput experimental approach to identify miRNA targets in human cells. Nucleic acids research. 2009; 37:e137.
2. Livak KJ and Schmittgen TD. Analysis of relative gene expression data using real-time quantitative PCR and the 2(-Delta Delta C(T)) Method. Methods. 2001; 25:402-408.
3. Eberwine J, Yeh H, Miyashiro K, Cao Y, Nair S, Finnell R, Zettel M and Coleman P. Analysis of gene expression in single live neurons. Proceedings of the National Academy of Sciences of the United States of America. 1992; 89:3010-3014.
4. Varemo L, Nielsen J and Nookaew I. Enriching the gene set analysis of genome-wide data by incorporating directionality of gene expression and combining statistical hypotheses and methods. Nucleic acids research. 2013; 41:4378-4391.

SUPPLEMENTARY FIGURES AND TABLES

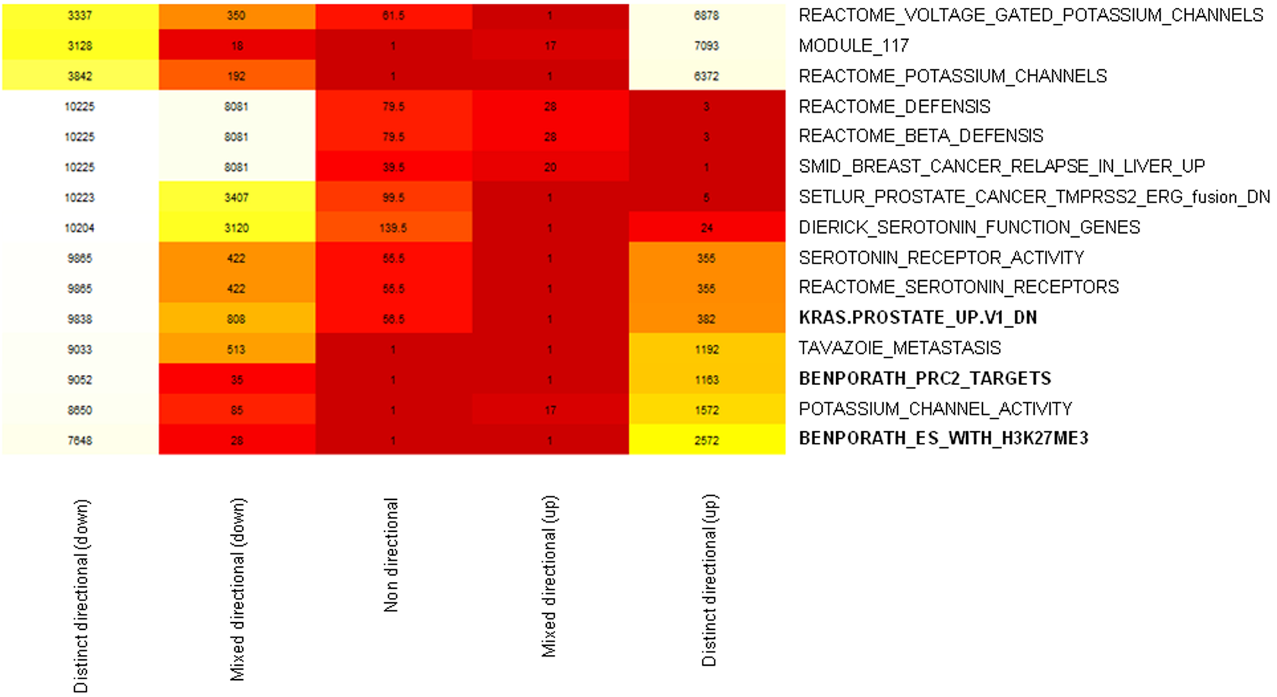

Supplementary Figure S1: Gene set analysis (GSA) of Ago-IP$_{IgG}$ using the PIANO algorithm [4]. Gene sets with low ranks in the "Non directional" class were defined as highly regulated. Additional low ranks in the "Mixed directional (up)" and "Distinct directional (up)" classes indicated that subsets or all genes of the gene set were significantly enriched in Ago-IP$_{IgG}$. Red represents low ranks and white high ranks. For detailed information see Varemo et al. [4].

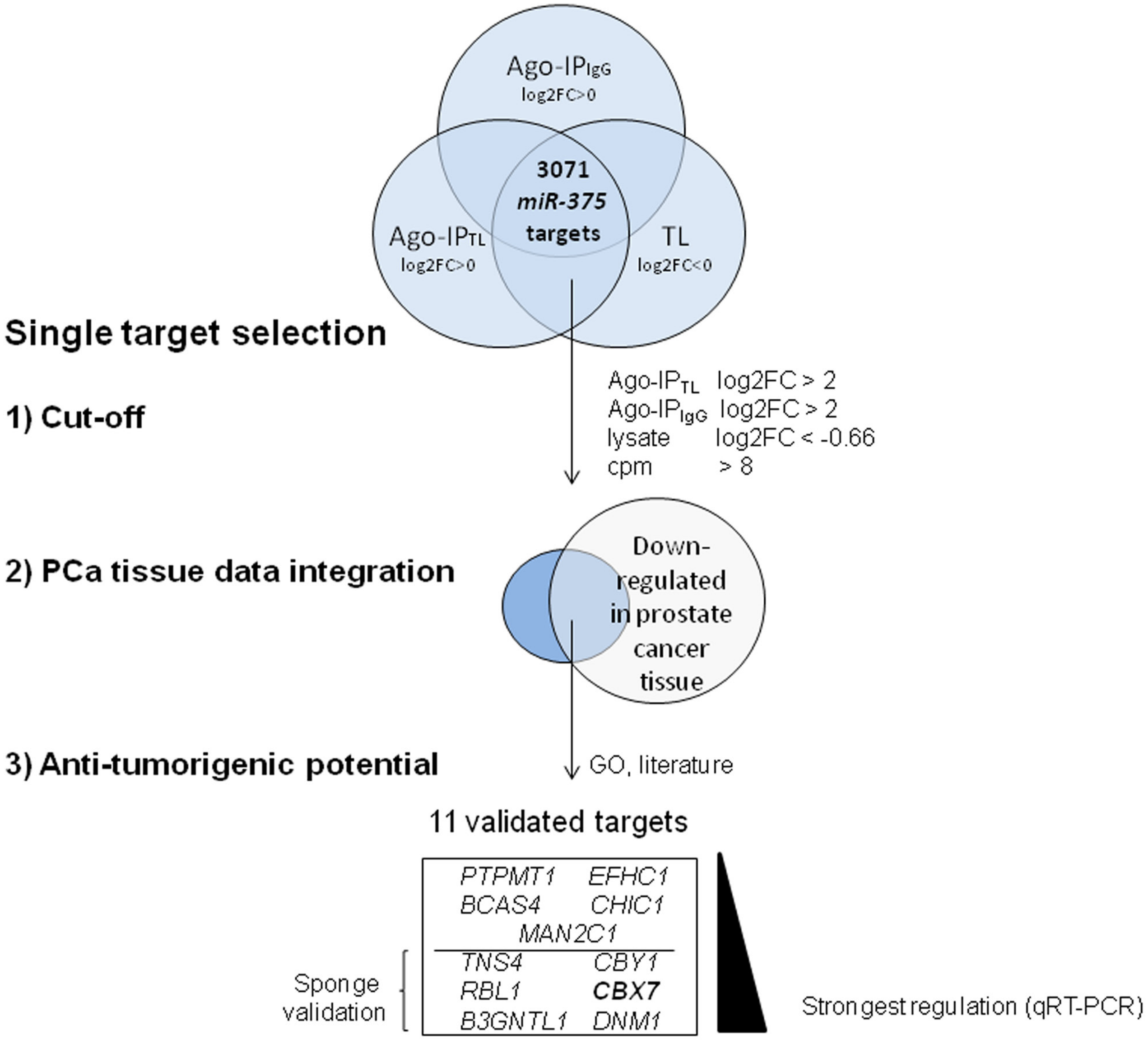

Supplementary Figure S2: Workflow of *miR-375* target filtering. cpm = counts per million reads.

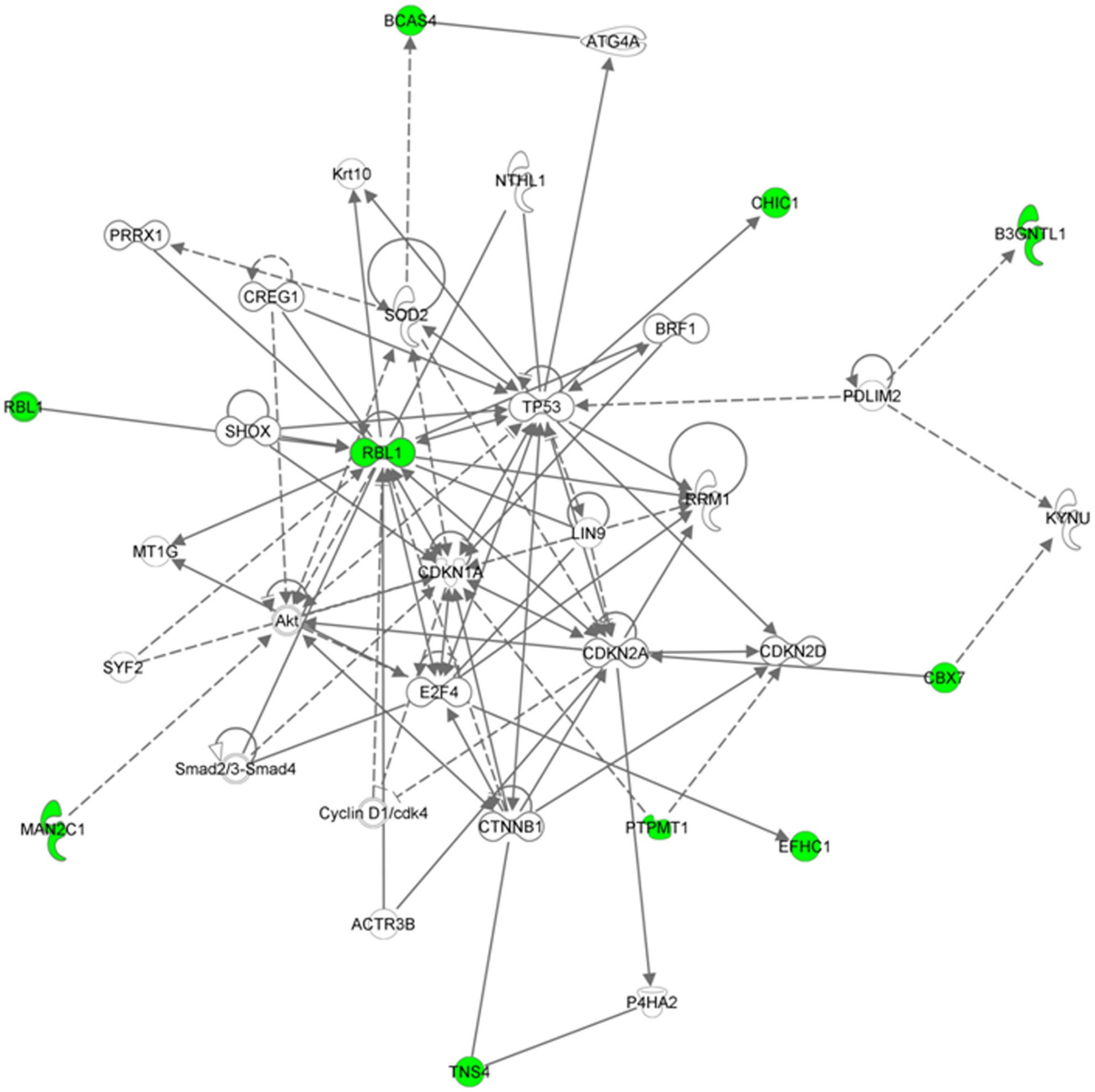

Supplementary Figure S3: Pathway analysis of the eleven validated *miR-375* target genes. Nine of eleven (highlighted in green) were assigned to the network "cell cycle, cellular development and cancer" in IPA analysis.

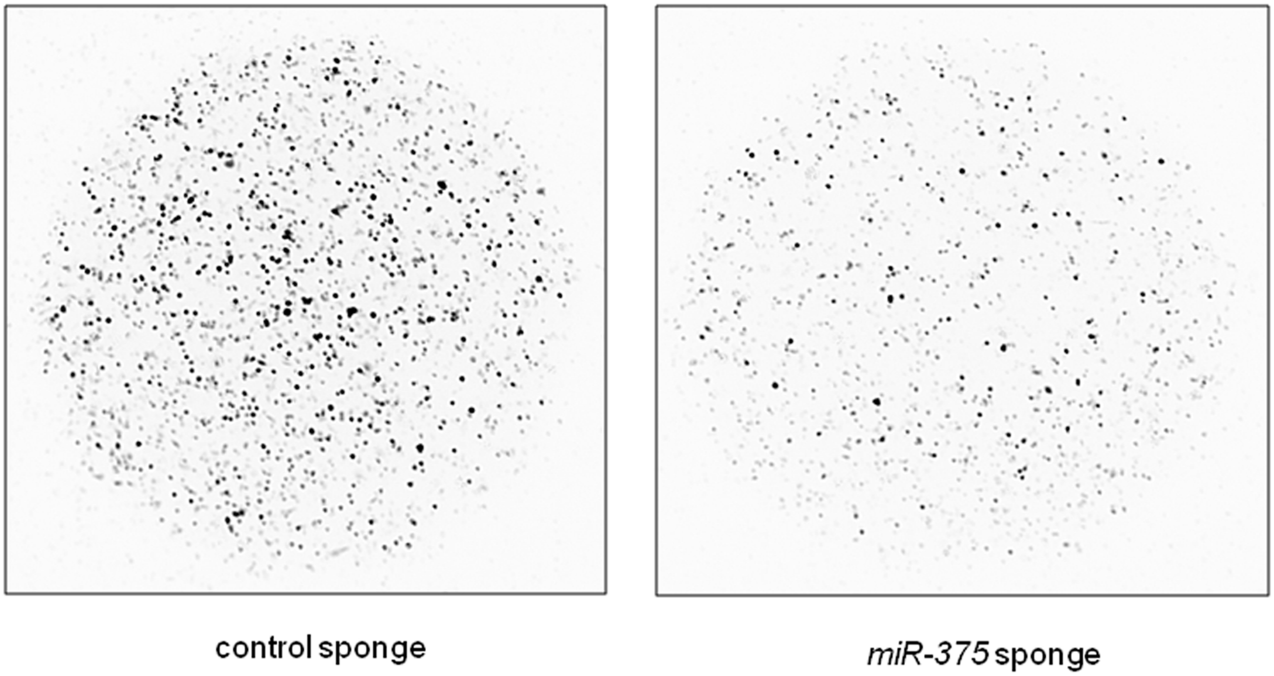

Supplementary Figure S4: PC-3 cells expressing GFP control sponge (left) and GFP *miR-375* sponge (right). Following sponge transfection, the GFP *miR-375* sponge signals were reduced by endogenous *miR-375* compared to the GFP control sponge signals after 48 h.

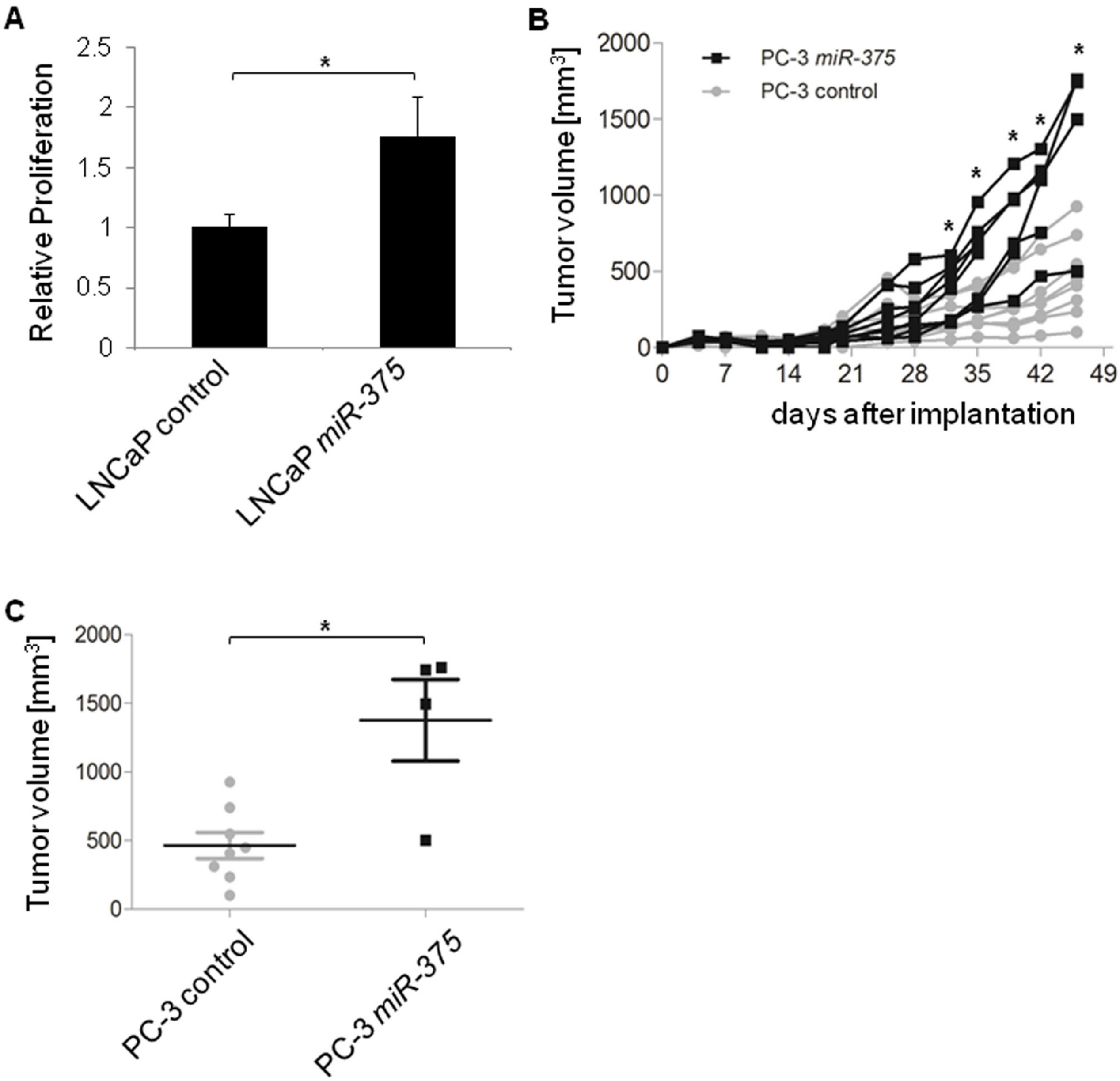

Supplementary Figure S5: A. *miR-375* overexpression enhances proliferation of LNCaP cells. Proliferation was measured 96 h after transfection. The experiment was performed in triplicates. *$P \leq 0.05$. Error bars show s.d.. **B** and **C.** Mouse xenograft model. (B), Growth of tumors in individual mice following implantation of PC-3 *miR-375* cells (black; n = 8) or PC-3 control cells (grey; n = 8). (C), Absolute tumor volumes 46 days after injection of PC-3 *miR-375* or PC-3 control mice. Error bars show s.e.m..

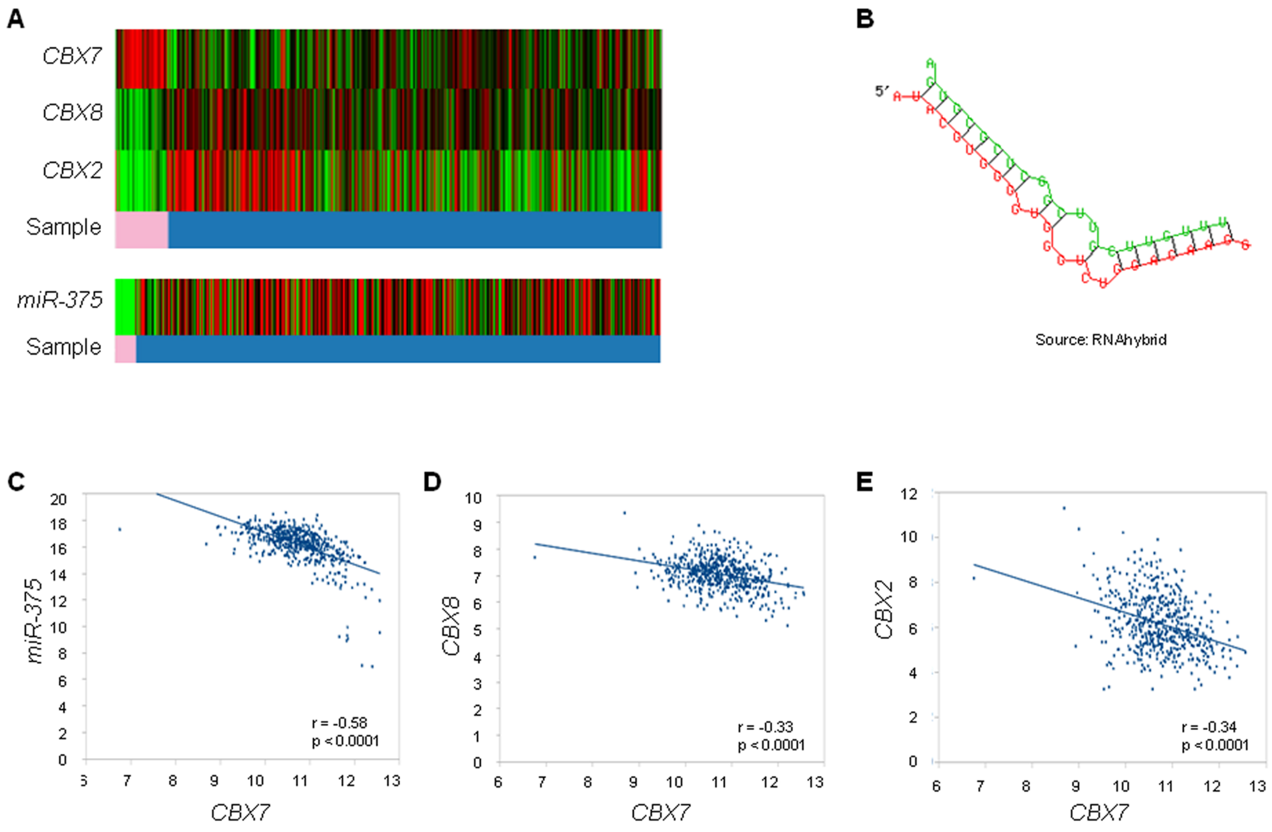

Supplementary Figure S6: A. Expression (red = high; green = low) of *CBX2, CBX7, CBX8* and *miR-375* in the TCGA prostate adenocarcinoma data set (n = 550 samples). Samples: pink = Normal, blue = Tumor. **B.** *MiR-375* binding site in the 3'-UTR of *CBX7*. **C-E.** Correlation analysis of *miR-375* and *CBX7* (C), *CBX7* and *CBX8* (D), and *CBX7* and *CBX2* (E), respectively (*r* calculated by Pearson's correlations).

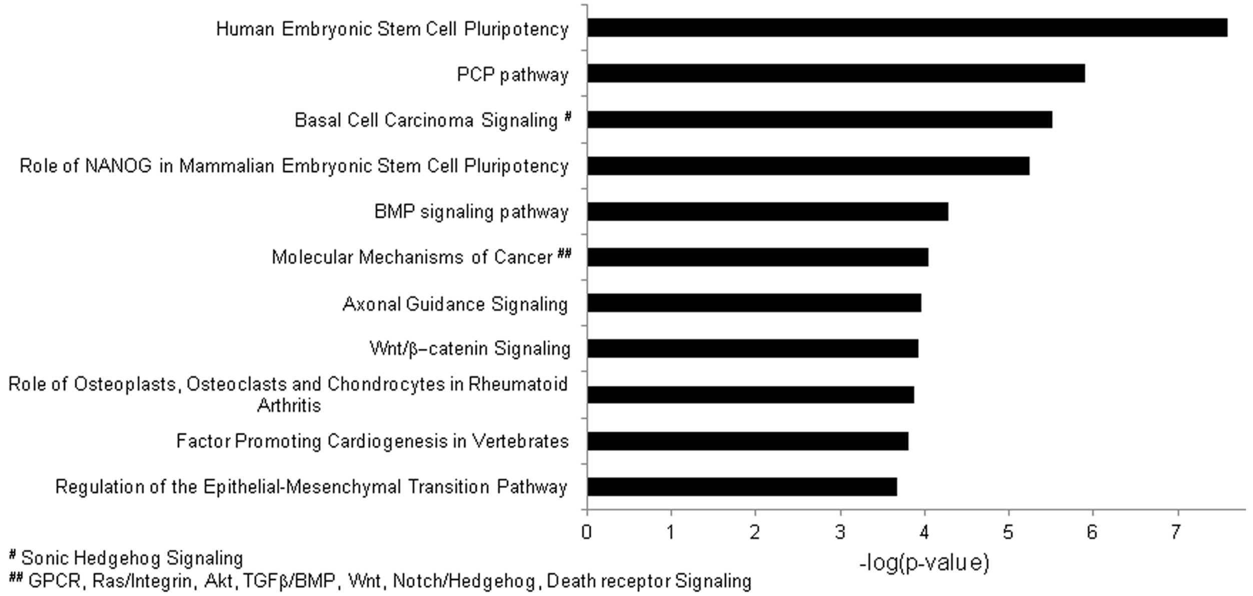

Supplementary Figure S7: Pathways associated with CBX7 target genes that are upregulated in PC-3 *miR-375* cells.

Supplementary Table S1: Number of reads per sample obtained by high-throughput sequencing

Sequencing Lane 1

Treatment	IP	Replicate	Index	Number of reads	Percentage of reads
miR-375	Ago	1	TGACCA	26,063,627	18.7
miR-375	IgG	1	GCCAAT	19,743,327	14.2
miR-375	TL	1	CTTGTA	28,304,273	20.3
control	Ago	1	CGATGT	21,699,896	15.6
control	IgG	1	ACAGTG	21,165,623	15.2
control	TL	1	CAGATC	18,922,485	13.6
undetermined			-	3,488,009	2.5

Sequencing Lane 2

Treatment	IP	Replicate	Index	Number of reads	Percentage of reads
miR-375	Ago	2	TGACCA	28,518,397	18.2
miR-375	IgG	2	GCCAAT	25,108,314	16.0
miR-375	TL	2	CTTGTA	23,353,731	14.9
control	Ago	2	CGATGT	28,344,485	18.1
control	IgG	2	ACAGTG	24,964,036	15.9
control	TL	2	CAGATC	22,225,208	14.2
undetermined			-	4,029,911	2.6

Sequencing Lane 3

Treatment	IP	Replicate	Index	Number of reads	Percentage of reads
miR-375	Ago	3	TGACCA	23,402,781	16.5
miR-375	IgG	3	GCCAAT	23,622,331	16.6
miR-375	TL	3	CTTGTA	23,567,129	16.6
control	Ago	3	CGATGT	22,506,797	15.8
control	IgG	3	ACAGTG	23,700,242	16.7
control	TL	3	CAGATC	21,499,494	15.1
undetermined			-	3,888,692	2.7

Supplementary Table S2: Differentially expressed genes in RNA-Seq of PC-3 cells overexpressing *miR-375*.

See Supplementary File 1

Supplementary Table S3: Potential direct *miR-375* targets identified by the integration of Ago-RIP-Seq and RNA-Seq data. *MiR-375* targets were defined by positive log2FC values in Ago-IP_{IgG} and Ago-IP_{TL} and negative log2FC values in lysate.

See Supplementary File 2

Supplementary Table S4: Direct *miR-375* targets (4-fold enrichment in Ago-IP_{IgG} and Ago-IP_{TL} as well as downregulation by a factor of at least 1.5 in lysate and > 8 cpm). Up- and downregulated genes in prostate cancer tissues are indicated by "up", and "down", respectively (GSE29079). T = tumor, N = normal.

See Supplementary File 3

Supplementary Table S5: CBX7 absence in lymph node metastases was independent from the CBX7 status of the primary prostate cancer samples of the same patients

	CBX7 expression in primary tumor; n[%]		
**CBX7 expression in lymph nodes; n[%]**	**Positive**	**Negative**	**Total**
**Positive**	9[15.5]	8[13.8]	17[29.3]
**Negative**	18[31.0]	23[39.7]	41[70.7]
**Total**	27[46.6]	31[53.4]	58[100.0]

p = 0.530, Pearson Chi^2 test, two-sided

Supplementary Table S6: Differentially expressed genes in microarray analysis following *CBX7* knockdown in LNCaP cells.

See Supplementary File 4

Supplementary Table S7: Sequences of primers and siRNAs.

See Supplementary File 5
